# Drowning prevention in the Americas: current and future opportunities arising from the first WHO Global Status Report on Drowning Prevention

**DOI:** 10.1016/j.lana.2023.100585

**Published:** 2023-09-06

**Authors:** Ana Catarina Queiroga, Ricardo Pérez-Núñez

**Affiliations:** PAHO – Pan-American Health Organization, Washington, DC, USA

Unintentional drowning is among the 5 leading causes of death, premature mortality, and a major contributor to disability in children, adolescents, and youth in the Region of the Americas. The WHO's Region of the Americas includes 35 Member States and four Associate Members, encompassing a wide range of socio-geo-demographic and economic settings, resulting in significant differences in drowning related risk factors and burden. From 2000 to 2019, age-standardized death rates per 100,000 population due to unintentional drowning decreased in all subregions, except in the Non-Latin Caribbean, where the rate increased 22.6% from 3.2 to 3.9^1^ (Table 1). Regional level data conceals the heterogeneity of drowning burden and context. Different exposures and interactions with water as well as the varying levels of coverage and implementation of preventive strategies might be the key factors for the observed varying death rates due to drowning from 18.5 in Guyana to 0.3 in Jamaica. Understanding the scope of existing drowning prevention efforts and identifying key stakeholders to engage in such work are critical for effective and coordinated action. Currently, a worldwide effort is being undertaken to collect data to produce the first Global Status Report on Drowning Prevention (GSRDP). It is expected that the reasons for such a high difference in drowning mortality among member states will be clarified once the upcoming report is published by the end of 2024.

**Table 1:** Unintentional drowning age-standardized death rate per 100,000 in the Region of the Americas and by Subregion.

Location	Drowning death rate per 100,000 (UI range)		PC^a^
	2000	2019	
Region of the Americas	2.62 (2.18–3.17)	1.76 (1.38–2.24)	32.8
Andean area	3.17 (2.32–4.24)	1.81 (1.11–2.79)	42.9
Central America, Mexico and Latin Caribbean	3.76 (2.83–4.97)	2.24 (1.44–3.32)	40.4
North America	1.22 (1.13–1.32)	1.19 (1.09–1.29)	2.5
Non-Latin Caribbean	3.23 (2.42–4.21)	3.96 (2.73–5.53)	−22.6
Southern Cone and Brazil	3.41 (3.04–3.82)	2.05 (1.81–2.32)	39.9

a Percentage Change of the drowning age-standardized death rate between 2019 and 2000.

A recent scoping exercise of drowning prevention efforts in the region (PAHO, unpublished), gathering detailed information about Argentina, Bahamas, Bolivia and Guyana, showed that existing efforts to take action on drowning prevention in these countries are in line with WHO recommended guidance. However, despite some policy support, generally these efforts have not been implemented systematically at national level. Barriers to progress further are the lack of consistent and timely data collection (particularly on characterization of the types of drownings, exposure to risk factors, and non-fatal drowning events), instability of the funding sources, sparse program evaluations and the lack of strategic planning for long-term implementation of drowning prevention interventions.

The adoption of the first UN Resolution on Global Drowning Prevention^2^ and the World Health Assembly (WHA) Resolution–*Accelerating action on global drowning prevention*^3^ is a clear recognition that drowning is a public health priority and that collaborative action is needed to halt its burden. Member States have committed to promote drowning prevention through community engagement, capacity building and international cooperation. The WHA resolution requests Member States to assess their national drowning situation, develop and implement related programming, and ensure policy planning across a wide range of sectors to reduce drowning risks.

PAHO as the regional office of WHO is currently working with Member States of the Americas to collect the information that will integrate the first GSRDP. A better understanding of the country specific magnitude of drowning, as well as the level of implementation of evidence-based interventions to prevent and respond to drowning will result from this effort. This will also serve as a baseline to monitor and evaluate the impact of the actions that will result from this global initiative. All this has the potential to support and inform national/regional drowning prevention strategies.

Opportunities to advance action to reduce the burden of drowning in the Americas that may be leveraged by the GSRDP include:

• designating a national lead agency or support the establishment of a multisectoral coordinative body.
  o Institutions from national data focal points officially appointed by countries to convene different stakeholders involved in drowning prevention could start taking the lead.
• documenting and sharing knowledge gained in drowning prevention efforts
  o Consensus meetings that will take place nationally to complete the GSRDP could be a starting point for this.
• performing a comprehensive analysis of existent national drowning prevention efforts, from legislation to educational programs and intervention plans—which is the spirit of the GSRDP
• improving availability and quality of drowning data reporting
  o GSRDP might provide useful information on the gaps in information available for each country.
• improving the enforcement mechanisms of laws and policies
• strengthening data systems that capture, analyze and validate data on drowning events
  o the GSRDP can be a tool to build a roadmap to guide local authorities on this.

The WHO's announcement of a Global Alliance to increase cohesion, and collaboration across UN Agencies, civil society and the donor community is a major opportunity for Member States to committing to develop a drowning prevention agenda in their countries by mobilizing resources for continued long-term funding, refining planning and multisectoral coordination, and ensuring that programs are evaluated for effectiveness.

## Contributors

ACQ and RPN conceptualized this work. ACQ drafted the first draft. All authors have provided critical revision to the text. All authors agree to submission of the final manuscript.

## Data sharing statement

Not applicable.

## Declaration of interests

The authors declare no conflicts of interest.
